# Molecular nanoarchitectonics: unification of nanotechnology and molecular/materials science

**DOI:** 10.3762/bjnano.14.35

**Published:** 2023-04-03

**Authors:** Katsuhiko Ariga

**Affiliations:** 1 International Center for Materials Nanoarchitectonics (WPI-MANA), National Institute for Materials Science (NIMS), 1-1 Namiki, Tsukuba 305-0044, Japan; 2 Department of Advanced Materials Science, Graduate School of Frontier Sciences, The University of Tokyo, 5-1-5 Kashiwanoha, Kashiwa, Chiba 277-8561, Japan

**Keywords:** local probe chemistry, materials chemistry, nanoarchitectonics, nanotechnology, on-surface synthesis

## Abstract

The development of nanotechnology has provided an opportunity to integrate a wide range of phenomena and disciplines from the atomic scale, the molecular scale, and the nanoscale into materials. Nanoarchitectonics as a post-nanotechnology concept is a methodology for developing functional material systems using units such as atoms, molecules, and nanomaterials. Especially, molecular nanoarchitectonics has been strongly promoted recently by incorporating nanotechnological methods into organic synthesis. Examples of research that have attracted attention include the direct observation of organic synthesis processes at the molecular level with high resolution, and the control of organic syntheses with probe microscope tips. These can also be considered as starting points for nanoarchitectonics. In this review, these examples of molecular nanoarchitectonics are introduced, and future prospects of nanoarchitectonics are discussed. The fusion of basic science and the application of practical functional materials will complete materials chemistry for everything.

## Review

### Introduction

Nanotechnology is a game changer that has innovated the course of scientific research. Nanotechnology innovations have unlocked mysteries at the nanoscale [1–3]. These research innovations have bridged the gap between nanoscale basic science and materials properties. The development of functional materials is an important key to solving various problems in human society. Functional materials have been created together with developments of well-established science such as organic chemistry [4–6], inorganic chemistry [7–9], polymer chemistry [10–12], supramolecular chemistry [13–15], coordination chemistry [16–18], other materials chemistry [19–21], and bio-related chemistry [22–24]. Accordingly, it has become clear that precise control of structures is necessary to improve functionality [25–26]. With the development of nanotechnology, this has come to be explained more rationally. Nanotechnology has a significant impact not only on the extreme science of the very small size regions [27–29], but also on realistic materials science [30–32].

For example, the elucidation of catalytic sites exhibiting very high activity [33–35], efficient molecular identification [36–38], and device switching mechanisms based on nanoscale phenomena [39–41] have been revealed. The contribution of nanotechnology is not limited to the elucidation of such physical properties of materials. Nanotechnology has also contributed greatly to the elucidation of the elementary processes of materials synthesis. The development of observation and analysis techniques for ultrasmall structures by nanotechnology has clarified processes of materials creation. For example, as summarized in a recent review by Harano, electron microscopic imaging at the atomic and molecular level has revealed the self-assembly mechanisms of crystal nuclei in organic crystals and metal-organic frameworks [42]. It has become possible to obtain statistical information on the size and structure of individual prenucleation clusters that cannot be examined by conventional analytical methods. It is also possible to reveal how functional sites such as catalysts are incorporated into the immobilization process. For example, an atomistic understanding of the structure of heterogeneous catalysts consisting of MoO_2_ complexes on carbon nanohorns has been reported [43]. Bottom-up synthesis of materials using molecular and ionic units, which is widely used in supramolecular chemistry and coordination chemistry, is now being elucidated by nanotechnology under observation of actual materials. Thus, the contribution of nanotechnology to the creation of materials cannot be ignored.

**Figure 1:**
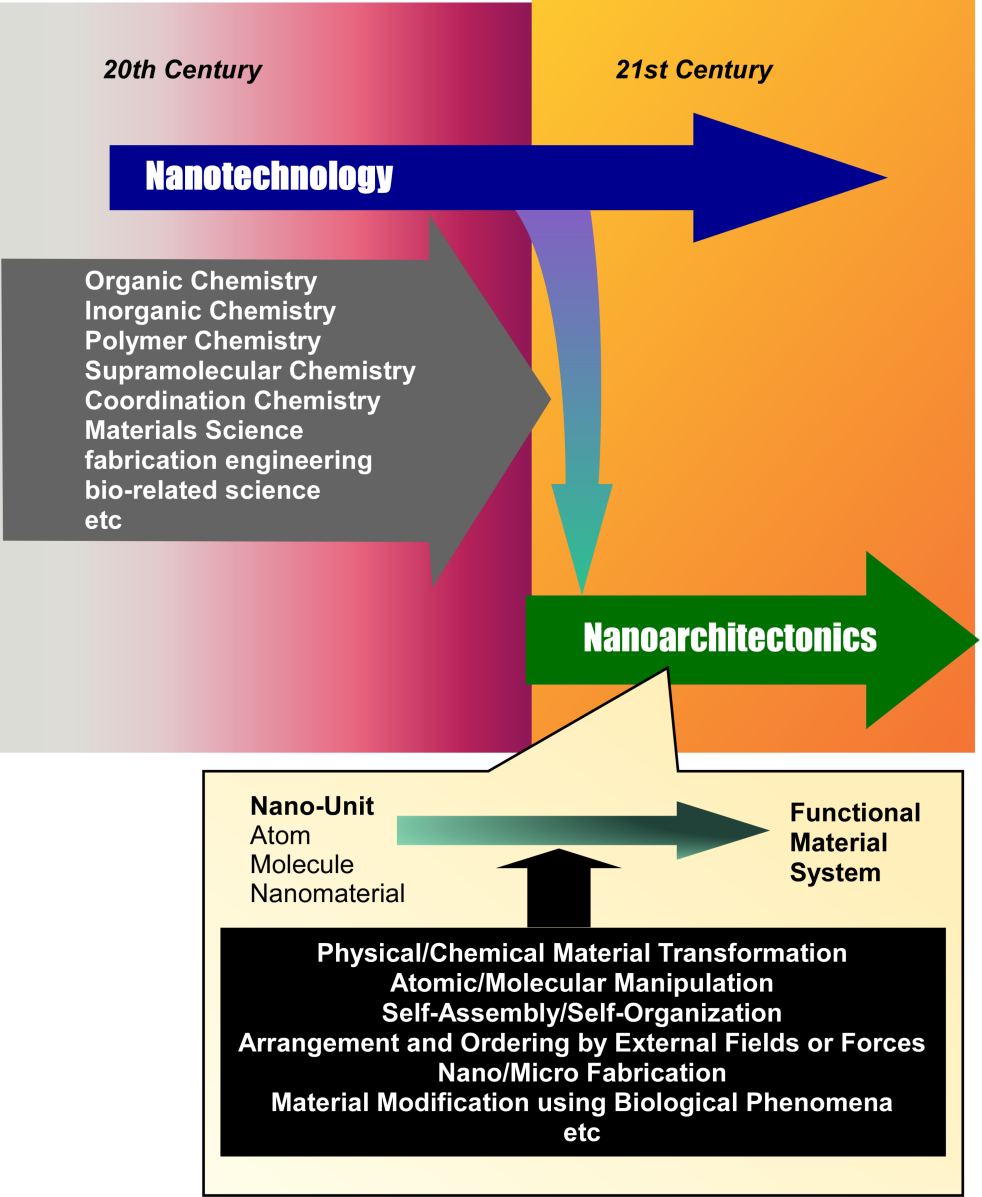
History and outline of the nanoarchitectonics concept.

The development of nanotechnology has provided an opportunity to integrate a wide range of phenomena and disciplines from the atomic scale, the molecular scale, and the nanoscale to the materials level. Under these circumstances, a unifying concept becomes important. Nanoarchitectonics [44–45], which can be considered a post-nanotechnology concept [46], can play this role. Just as Richard Feynman proposed nanotechnology in the 20th century [47–48], nanoarchitectonics was proposed by Masakazu Aono in the early 21st century [49–50]. Nanoarchitectonics is a methodology for creating functional materials systems using nanoscale units such as atoms, molecules, and nanomaterials. Nanoarchitectonics also integrates nanotechnology with other research fields such as organic chemistry, inorganic chemistry, polymer chemistry, supramolecular chemistry, coordination chemistry, materials science, fabrication engineering, and bio-related science (Figure 1) [51–52].

Materials synthesis by nanoarchitectonics is envisioned to integrate and use various processes [53]. For example, physical/chemical material transformation, atomic/molecular manipulation, self-assembly/self-organization, arrangement and ordering by external fields or forces, nano/microfabrication, and material modification using biological phenomena are selected and combined. These processes include both equilibrium and nonequilibrium processes. The structures created by combining these processes or by working on them step by step are asymmetric and hierarchical. Compared to structures created by self-assembly based on simple equilibrium processes, more hierarchical and complex structures are obtained in nanoarchitectonics processes [54]. In addition, several uncertainties in the nanoscale world from which architectures are derived have a non-negligible effect. For example, thermal fluctuations, stochastic distributions, and quantum effects affect nanoscale phenomena. Therefore, when building materials from nanoscale units, these uncertainties are included to harmonize the various effects [55–56]. Recent publications advocating nanoarchitectonics show that it is widely applied in academic fields such as materials synthesis [57–59], structural control [60–63], physical phenomena [64–66], and basic biochemistry [67–69], as well as in applied fields such as catalysis [70–72], sensors [73–75], devices [76–78], energy [79–81], environment [82–85], and medicine [86–89]. Since all the materials are composed of atoms and molecules, nanoarchitectonics is considered a universal unified concept that can be applied to all targets. Therefore, it can be considered as the method for everything in materials science [90], analogous to the theory of everything in physics [91].

Nanoarchitectonics is a highly universal concept. Therefore, the fabrication of structures from molecules to materials that has been done so far can also be considered part of the nanoarchitectonics processes. Here, some of the recent examples are presented. Molecular synthesis of unusual structures can lead to novel functional structures. Segawa's recent review describes the synthesis of nonplanar structures by molecular nanoarchitectonics of sp^2^-hybridized carbon atoms [92]. The flexibility of the structure allows a wide variety of nonplanar aromatic hydrocarbons to be built and highly distorted structures to be synthesized. These nonplanar aromatic hydrocarbons are expected to have a wide range of applications, including semiconductors, light-emitting devices, bioimaging, and pharmaceuticals. As a polymer nanoarchitectonics, Kubo and co-workers have developed room-temperature phosphorescence-active thiophene boronate ester-crosslinked polyvinyl alcohol films [93]. The films are capable of multicolor afterglow emission by changing the amount of sulforhodamine B. Molecular design and nanoarchitectonics can sometimes yield unusual physical properties. A recent review by Tsuji reports the realization of cryogenic properties at room temperature by chemical immobilization of molecular structures [94]. Functions such as optoelectronic properties and bioactivity of materials strongly depend on molecular structures. Controlling macroscopic functions by controlling the molecular structure is a very well-established design strategy in molecular nanoarchitectonics.

There is also a strong research interest in combining structure-forming techniques to develop functional materials. For example, the Langmuir–Blodgett (LB) method [95–96] and layer-by-layer (LbL) assembly [97–98] are used to organize materials from molecules. Oishi and co-workers have used the LB method to create layered perovskite films with a uniform surface [99]. Similarly, LbL assembly is a simple and convenient way to create a wide range of materials in a designed layered structure. As summarized in the review by Akashi and Akagi, LbL assembly is valuable as a pathway to the design and development of innovative biomaterials for tissue engineering [100]. Interfacial phenomena contribute significantly to material accumulation and property expression through nanoarchitectonics. Shi and co-workers created nanoparticle surfactants at liquid–liquid interfaces by exploiting the interaction between nanoparticles and polymer ligands [101]. They showed that a size-dependent aggregation of nanoparticle surfactants can be generated at the interface. Functional exploration through material accumulation and organization has also been widely conducted. The manipulation of precise molecular alignments and photochemical properties through multiple electrostatic interactions with two-dimensional clay mineral nanosheets has been summarized by Ishida [102]. Yue and Gong discuss the organization of gels [103]. Specifically, they outline a unique anisotropic hydrogel consisting of uniaxially aligned lamellar bilayers in an amorphous gel matrix. This gel organization exhibits a beautiful structural color that is sensitive to mechanical and chemical stimuli. The water impermeability of the bilayer membrane causes one-dimensional swelling and diffusion, and the hydrophobic aggregates act as sacrificial bonds to yield high mechanical strength and toughness during deformation.

The abovementioned general examples could be involved in parts of nanoarchitectonics processes. Nanoarchitectonics has the potential to encompass a very broad range of materials chemistry. Roughly categorizing these two categories, molecular nanoarchitectonics [104–106], which is architecture at the molecular level, and materials nanoarchitectonics [107–108], which is architecture at the materials level, are the two most important areas of nanoarchitectonics. Molecular nanoarchitectonics has recently been strongly promoted by incorporating nanotechnological methods into organic synthesis. Examples of research that have attracted attention include the direct observation of the organic synthesis process at the molecular level with high resolution, and the control of organic synthesis with probe microscope tips. These examples are the fusion fields of nanotechnology and organic synthesis. This can also be considered as a starting point for nanoarchitectonics. In this review, examples of molecular nanoarchitectonics are introduced, and future prospects of nanoarchitectonics are discussed.

### Nanotech-driven synthetic nanoarchitectonics

Molecular nanoarchitectonics is available on various size scales. Nanoarchitectonics at the molecular level involves the integration of synthetic organic chemistry and nanotechnology. For example, organic synthesis using probe microscope tips, a tool of nanotechnology, is now being realized [109–110]. Organic chemistry for single molecules on surfaces is being developed, which is different from the conventional organic synthesis in solution systems. In this section, organic synthesis induced by probe microscopy and related examples are presented. Although these studies have not been always called nanoarchitectonics, their content deserves to be considered molecular nanoarchitectonics.

Okawa and Aono fabricated nanowires of polydiacetylene by creating a self-assembled monolayer of a diacetylene compound (10,12-nonacosazinoic acid) adsorbed on a graphite surface and biased with a scanning tunneling microscope probe [111]. By positioning the probe at a specific site, the polymerization of the chains was induced within defined small regions, and the propagation of a linear polymerization reaction was successfully initiated and terminated at an arbitrary point. In other words, the polymerization reaction can be triggered at a desired position on the surface. If nanoscale processing and connections can be precisely controlled in this way, molecular nanoelectronics beyond current silicon-based device technologies can be realized. It also provides new scientific opportunities, such as measuring electrical conduction in structurally perfect one-dimensional materials and analyzing the propagation mechanism of chain polymerization. Thus, various new types of physics and chemistry at the nanoscale could be developed.

**Figure 2:**
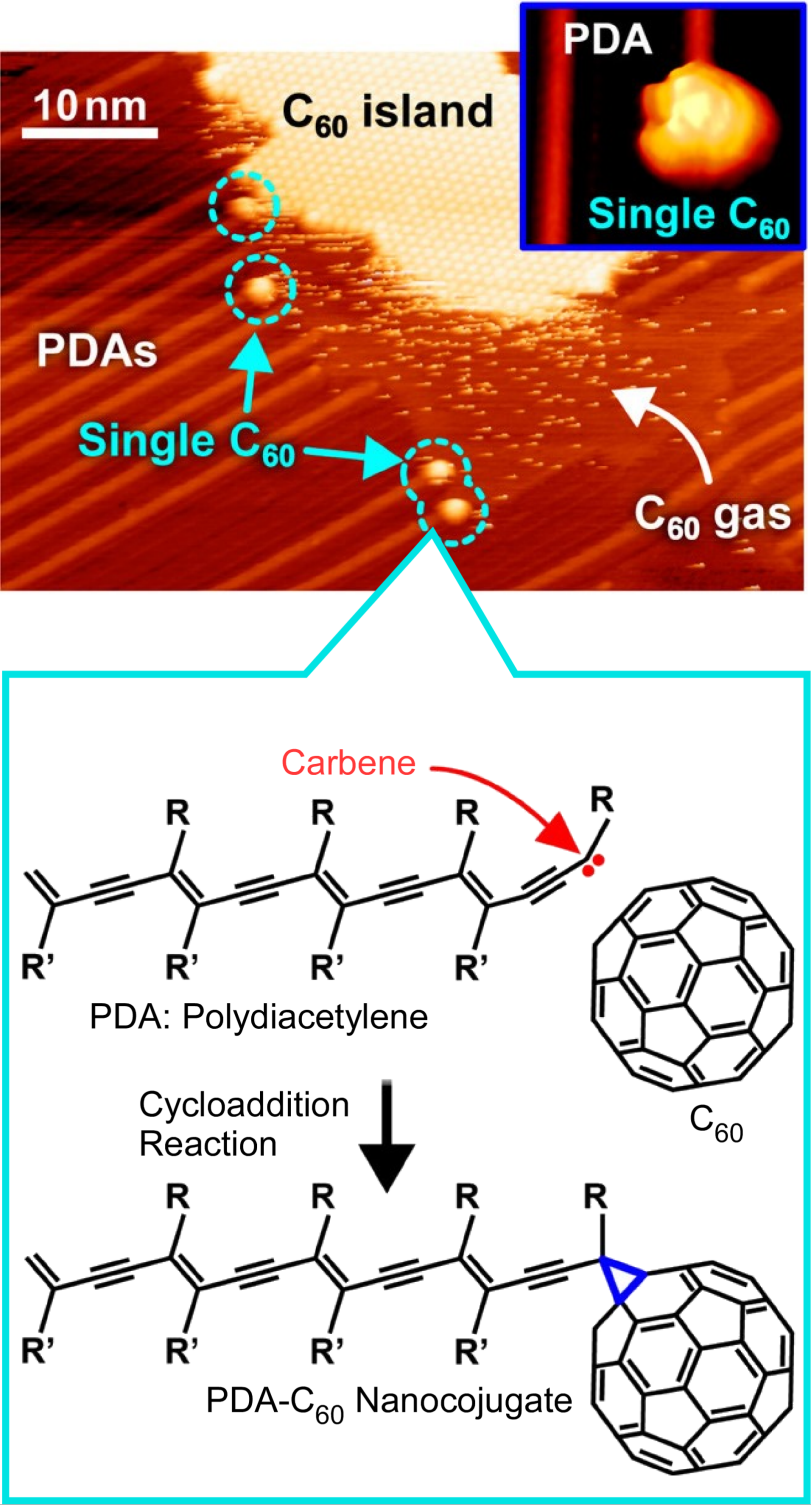
Fabrication of molecule–polymer nanoconjugates consisting of C_60_ molecules and polydiacetylene nanowires at designated locations on a solid surface. Figure 2 was adapted with permission from [112], Copyright 2014 American Chemical Society. This content is not subject to CC-BY-4.0.

Furthermore, such conductive polymer wires can be covalently linked to other functional components. Covalent bonding of functional molecules and conductive polymers to synthesize molecular composites at designated positions on a solid substrate could be a key technology for building nanoscale electronic circuits. Nakayama and co-workers have succeeded in controlling the self-assembly and intermolecular chemical reactions of functional molecular components predeposited on a solid surface [112]. Specifically, they fabricated molecule–polymer nanoconjugates consisting of C_60_ molecules and polydiacetylene nanowires at designated locations on a solid surface (Figure 2). First, diacetylene monomers were self-assembled on the solid substrate. Then, polydiacetylene nanowires were formed by chain polymerization between the monomer molecules by UV light irradiation. In the process, a cycloaddition reaction occurred between one nearby C_60_ molecule adsorbed on the surface and the most frontal part of the polydiacetylene molecular skeleton. As a result, nanojunctions were created. Scanning tunneling microscopy proved that the C_60_ molecule was covalently bonded to each end of the π-conjugated polydiacetylene backbone. The carbene at the front end of the polydiacetylene chain reacted with the 6-6 moiety of the C_60_ molecule, cleaving a double bond of the C_60_ molecule and forming a three-membered ring via cycloaddition as an intermolecular bond. Such an attempt is a promising approach to realize molecular nanoelectronics using molecule–polymer nanojunctions.

Molecular nanoarchitectonics of single molecule heterowires of conducting polymers has also been reported. Sakaguchi et al. realized the synthesis of molecular heterowires by a multistep electrochemical epitaxial polymerization technique [113]. This technique consists of combining two electrochemical polymerization processes using different monomer solutions. First, a voltage pulse was applied to an iodine-covered Au(111) substrate in an electrolyte solution containing the first thiophene monomer. This process produced the first polythiophene wires on the substrate. The substrate was then transferred to an electrolyte solution containing another thiophene monomer. The second process of applying voltage oxidized both the thiophene monomer in solution and the polythiophene on the substrate. This process resulted in the formation of heterowires in which the polymer initially formed on the substrate was linked to another type of thiophene polymer that was growing. In this way, nanoarchitectonics of conjugated polymers can be obtained on metal surfaces by multistep electrochemical epitaxial polymerization, controlling the length, density, bonding, and propagation direction of the molecules.

**Figure 3:**
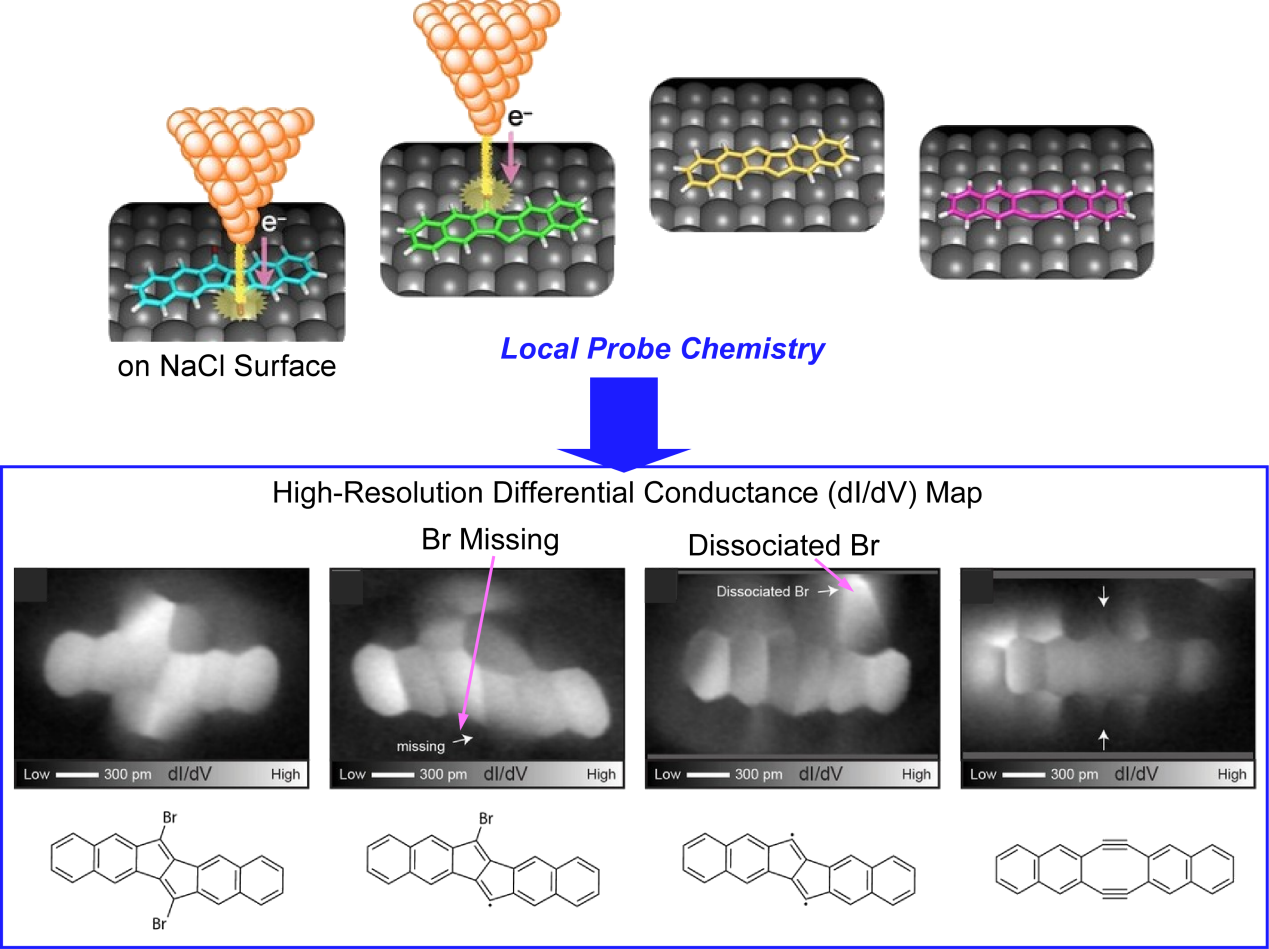
Local probe chemistry on an ultra-thin NaCl film formed on a Cu(111) surface for the synthesis of 6,13-dibromopentaleno[1,2-*b*:4,5-*b*′]dinaphthalene to a single Sondheimer–Wong diyne. Figure 3 was adapted from [114], S. Kawai et al., “An Endergonic Synthesis of Single Sondheimer–Wong Diyne by Local Probe Chemistry”, Angew. Chem., Int. Ed., with permission from John Wiley and Sons. Copyright © 2020 Wiley-VCH Verlag GmbH & Co. KGaA, Weinheim. This content is not subject to CC-BY-4.0.

Advances in surface scanning probe microscopy techniques have enabled the synthesis and analysis of unstable short-lived products by local probe synthesis as well as direct observation of molecular structures. Kawai et al. reported that local probe chemistry on an ultrathin NaCl film formed on a Cu(111) surface at 4.3 K led to the conversion of 6,13-dibromopentaleno[1,2-*b*:4,5-*b*′]dinaphthalene to a single Sondheimer–Wong diyne (Figure 3) [114]. The structures of the precursor, two intermediates, and the final product were identified in situ by differential conductance imaging using a CO-modified tip. The bias voltage was set above the lowest unoccupied molecular orbital energy and the probe was placed over the C–Br bond, which was then broken. After the reaction, a dip appeared on the side of the molecule where the debromination reaction occurred. Microscopic observation confirmed that the initial shape of the molecular skeleton was well preserved, but one bromine atom was clearly lost. Then the probe was placed over the second C–Br bond, and the sample bias voltage was swept to remove the second bromine atom. Close-up observation of the structure showed that the molecule was fully debrominated. Differential conductance imaging confirmed that the molecular skeleton, including the two naphthalene moieties, was clearly resolved. It was also observed that the two naphthalene moieties were slightly misoriented and split in the center of the skeleton. This molecular state was presumed to be a diradical. The authors concluded that the bond in the pentalene core was broken, and the resulting radical after the second bias sweep was converted to a single Sondheimer–Wong diyne. Unlike conventional solution synthesis, low-temperature local probe chemistry allows for a free control of the radical state. This kind of local probe chemistry as a synthesis technique opens up the possibility of nanoarchitectonics synthesis of carbon nanomaterials.

**Figure 4:**
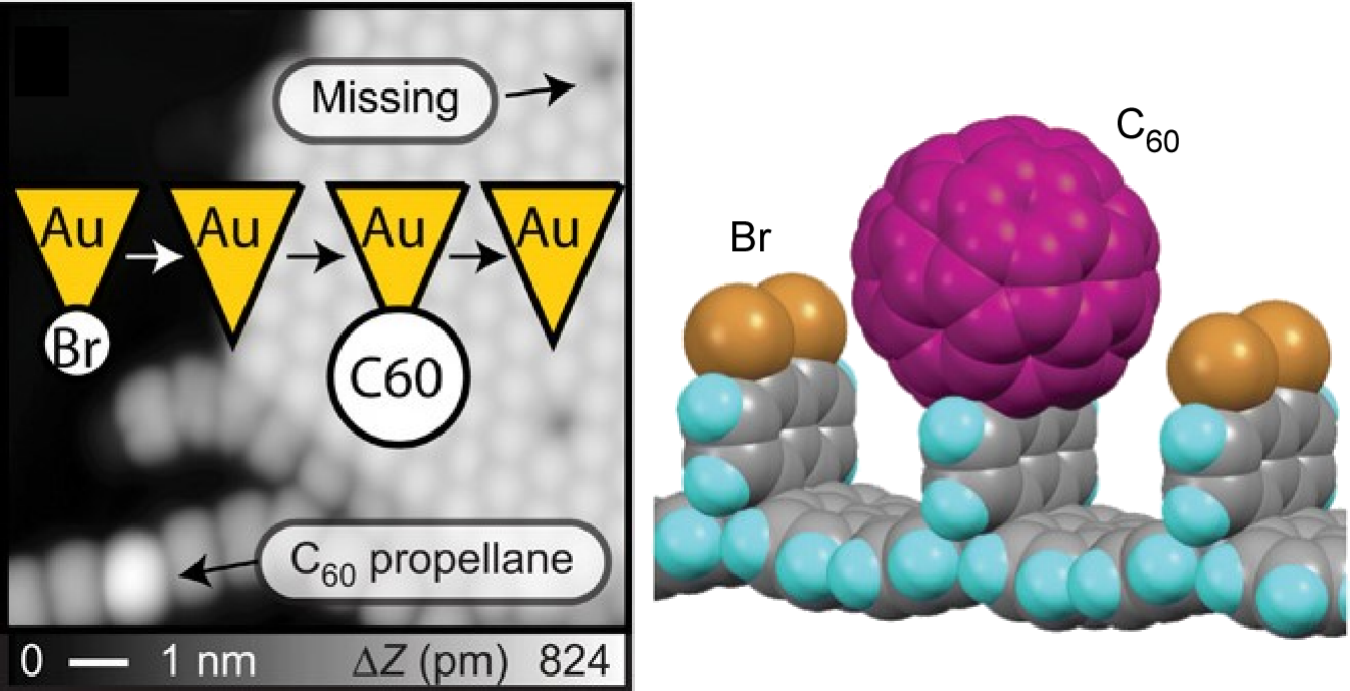
Local probe chemistry for covalent attachment of C_60_ fullerene molecule at demonstrated site on three-dimensional graphene nanoribbon. Figure 4 was reproduced from [115] (Copyright © 2020 S. Kawai et al., published by the American Association for the Advancement of Science, distributed under the terms of the Creative Commons Attribution 4.0 Non-commercial License, https://creativecommons.org/licenses/by-nc/4.0). This content is not subject CC-BY-4.0.

The tip-induced addition of single molecules was also realized. Kawai et al. synthesized three-dimensional graphene nanoribbons by surface chemistry and showed that local probe chemistry can be used to add different molecules by tip manipulation [115]. Specifically, they demonstrated that radicals created by tip-induced debromination can be reversibly terminated by C_60_ fullerene molecules (Figure 4). First, the Au probe was positioned at the target Br atom site. Then, when the bias voltage was swept, an abrupt change in the tunneling current was detected. As a result, the bromine atoms disappeared from the molecule and the C–Br bonds in the scanned region were easily broken. Next, when a C_60_ molecule was picked up from the surface with an Au tip and brought close to the radical at a low bias voltage, a large protrusion became clearly visible at the radical site. This indicated that the C_60_ molecule had been added to the radical site. In other words, a controlled addition reaction in a single molecule adsorbed on a surface by a local probe at low temperature was demonstrated. Long-lived radicals could be obtained, and C_60_ molecules could be selectively added to the sites. Such direct addition reactions enable the synthesis of single compounds at the atomic level. This is a breakthrough in organic chemistry and is drastically different from working principles of conventional solution synthesis. This is a coupling of nanotechnology and synthetic organic chemistry and can be regarded as a good example of molecular nanoarchitectonics.

**Figure 5:**
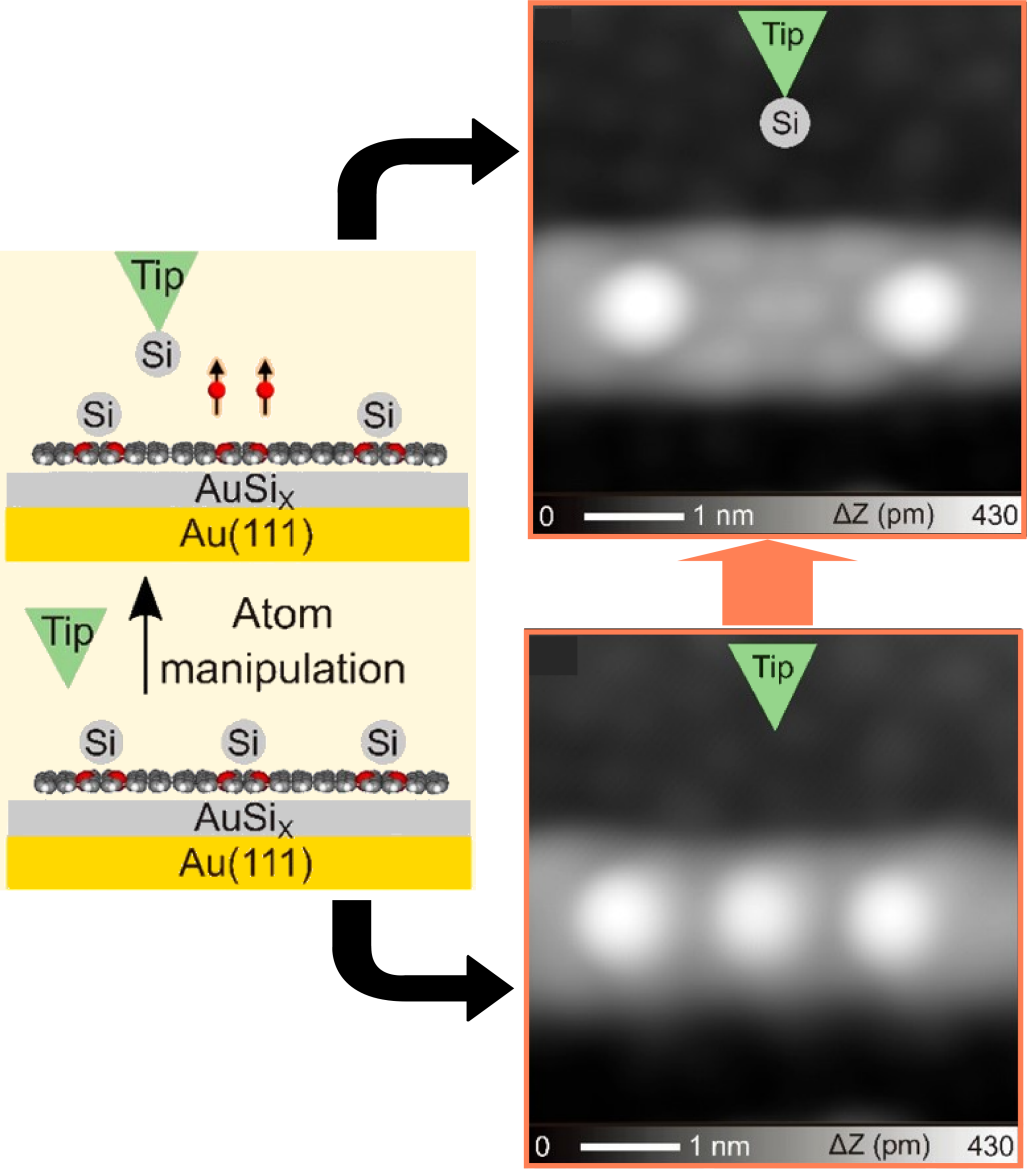
Selective removal of a Si atom by vertical manipulation with a tip. Figure 5 was adapted with permission from [116], Copyright 2022 American Chemical Society. This content is not subject to CC-BY-4.0.

Foster, Kawai, and co-workers have investigated the zero-bias peak at the center of an armchair-type graphene nanoribbon on a AuSi*_x_*/Au(111) surface using a combination of low-temperature scanning tunneling microscopy/spectroscopy and density functional theory calculations [116]. The zero-bias peak at the boron site embedded at the center of the graphene nanoribbon was investigated. Si atoms were removed by vertical manipulation with a tip (Figure 5). In this manipulation, the tip was positioned at a silicon site and then moved closer to the silicon atoms while recording the tunneling current. After the tip was brought close enough to obtain a single-atom conductance gap, it was retracted and silicon atoms were removed. A perpendicular magnetic field was applied to explore physical phenomena such as Kondo resonance. The nanoarchitectonics of magnetic topological states due to spin polarization in extended π-carbon systems is an important process for spintronics applications.

### On-surface synthetic nanoarchitectonics

Besides manipulating molecules and atoms with a tip, in situ observation of specific chemical reactions occurring on surfaces with a probe microscope is a very significant approach to molecular-level nanoarchitectonics. On-surface syntheses are products of the fusion of nanotechnology and synthetic organic chemistry [117–118], where molecular nanoarchitectonics comes into play. This section illustrates how various controlled structures can be obtained for objects such as graphene nanoribbons. Molecular nanoarchitectonics is an approach with great potential.

**Figure 6:**
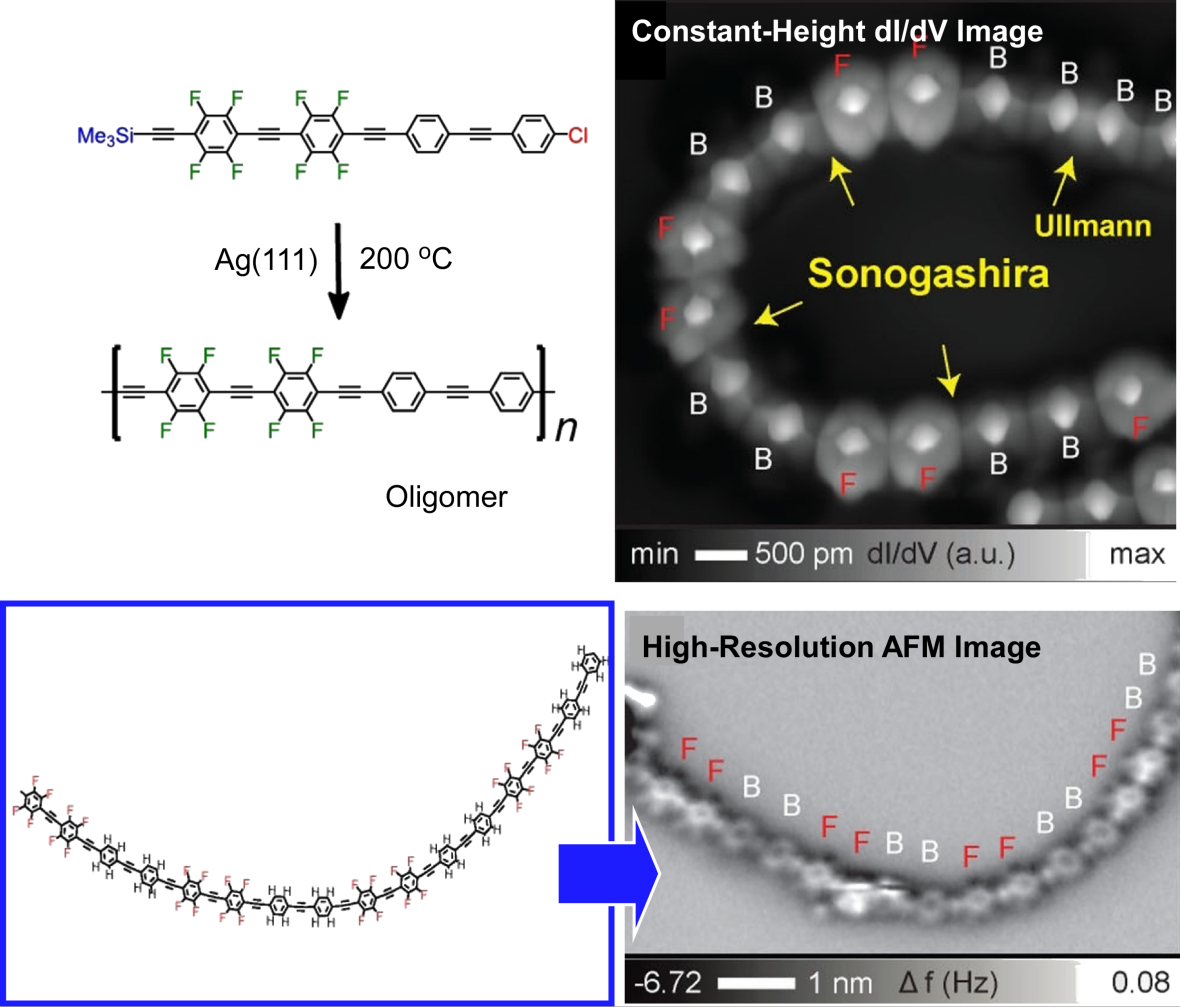
Chemoselective Sonogashira coupling between (trimethylsilyl)ethynyl and chlorophenyl groups on Ag(111) to create extended oligomer chain. Figure 6 was adapted from [119], K. Sun et al., “Head-to-Tail Oligomerization by Silylene-Tethered Sonogashira Coupling on Ag(111)”, Angew. Chem., Int. Ed., with permission from John Wiley and Sons. Copyright © 2021 Wiley-VCH GmbH. This content is not subject to CC-BY-4.0.

Orita, Kawai, and co-workers demonstrated controlled synthesis of conjugated oligomers by chemoselective Sonogashira coupling between (trimethylsilyl)ethynyl and chlorophenyl groups on Ag(111) (Figure 6) [119]. In this reaction, oligomers are obtained by CH_3_–Si bond activation at 130 °C and subsequent tether removal at 200 °C to form an intermolecular silylene tether (–Me_2_Si–). The sample was further heated at 200 °C to accelerate silylene tether desorption and complete the desilylation Sonogashira coupling. As a result, the oligomeric chains were significantly elongated. High-resolution scanning tunneling microscope (STM) topography shows alternating bright twin spots, which correspond to phenylene and tetrafluorophenylene, respectively. A high-resolution atomic force microscope (AFM) image of an entirely elongated fine nanowire and the corresponding chemical structure are also shown. In this attempt, phenylene–ethynylene nanowires with alternating polymerization of phenylene and tetrafluorophenylene have been successfully synthesized while simultaneously imaging the molecules. Sonogashira reactions at the surface are expected to pave the way for further functional nanostructures.

**Figure 7:**
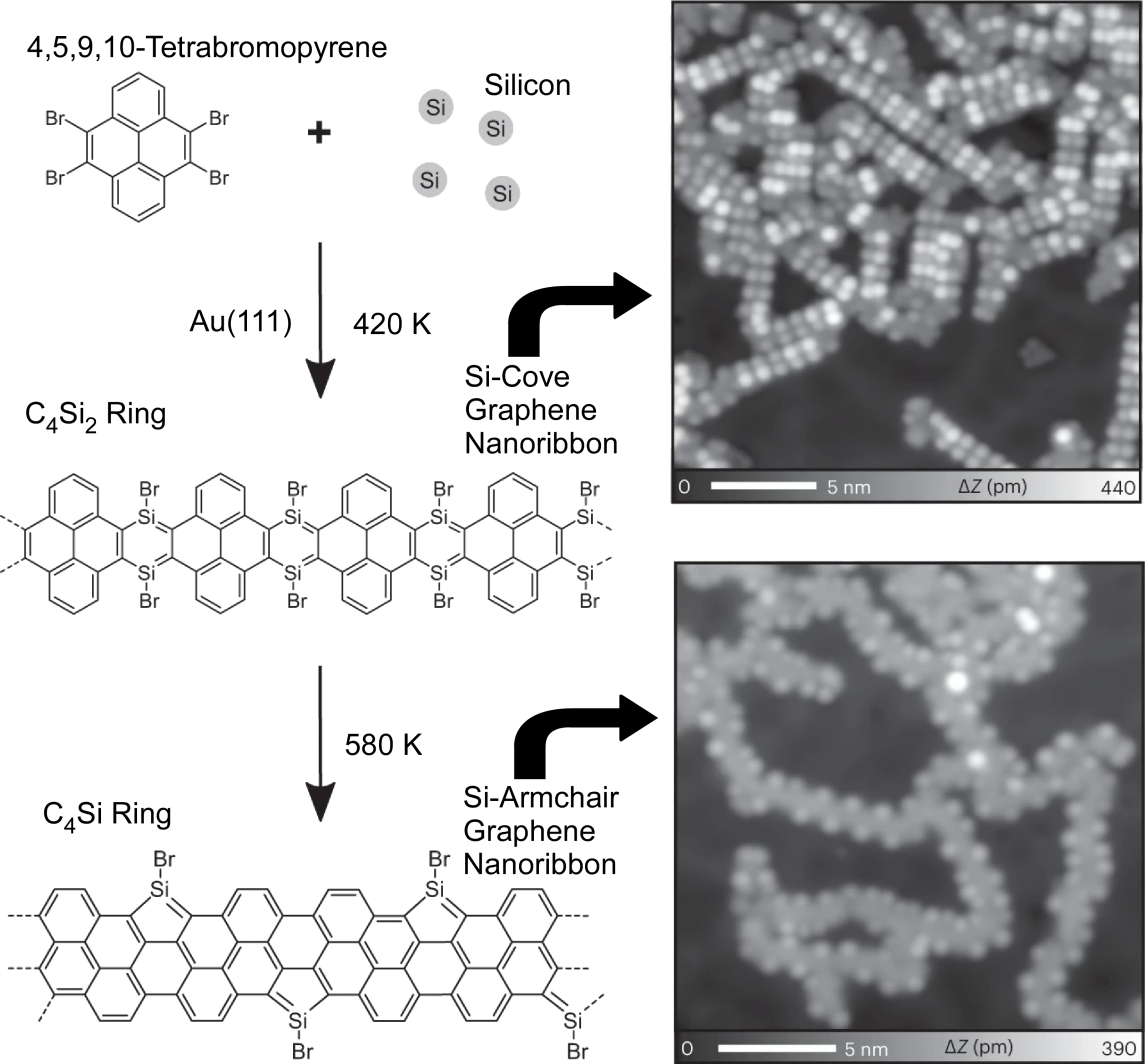
On-surface synthesis of silicon-substituted graphene-based materials. Figure 7 was adapted from [120] (© 2023 K. Sun et al., published by Springer Nature, distributed under the terms of the Creative Commons Attribution 4.0 International License, https://creativecommons.org/licenses/by/4.0).

In organic molecules and materials, the electronic structure and physical properties can be modified by replacing carbon with silicon. For example, silicon-substituted graphene-based materials exhibit exotic properties. However, it is difficult to synthesize silicon-substituted conjugated organic materials with atomic precision using conventional organic synthesis methods. Foster, Kawai, and co-workers have successfully synthesized one-dimensional and two-dimensional covalent organic frameworks on the surface of 1,4-disilabenzene (C_4_Si_2_) backbones (Figure 7) [120]. First, silicon atoms were deposited on a Au(111) surface and annealed to form an AuSi*_x_* film. Bromo-substituted polycyclic hydrocarbon precursors (triphenylene or pyrene) were then deposited on this surface and annealed to form a C_4_Si_2_ bridging network. In the linear structures obtained with pyrene precursors, the C_4_Si_2_ rings were converted to C_4_Si pentagonal siloles by further heat treatment. These results demonstrate that coupling nanoarchitectonics on C–Si surfaces is possible by depositing Si atoms and, subsequently, polycyclic hydrocarbons on Au(111). It is expected that various low-dimensional nanostructures will be synthesized by this on-surface synthetic nanoarchitectonics.

The bottom-up synthesis of graphene nanoribbons on surfaces has attracted much attention due to their high electronic, optical, and magnetic properties. Sakaguchi and co-workers have synthesized cove-shaped two-dimensional graphene nanoribbon networks by interconnecting one-dimensional self-assembled graphene nanoribbons on a Au(111) surface [121]. The structure of the two-dimensional graphene nanoribbon network consists of hybrid junctions of graphene nanoribbons of various widths, exhibiting both metallic and semiconducting properties. These networks have been applied to thermoelectric materials and have been found to exhibit low interplane thermal conductivity, which is not typical of carbon materials, while maintaining the interplane electrical conductivity. Müllen, Fuchs, Chi, and co-workers used 1,4,5,8-tetrabromonaphthalene as a molecular precursor and sequential dehalogenation reactions under mild conditions to synthesize very thin (five carbon atoms wide) armchair graphene nanoribbons on a Au(111) surface [122]. The spatial distribution of the electronic structure and other properties were investigated. Müllen, Fasel, and co-workers have succeeded in nanoarchitectonics of graphene nanoribbons with zigzag edges with atomic precision by on-surface synthesis via cyclodehydrogenation of precursor monomers [123]. The physical properties of the graphene nanoribbons, such as band structure, magnetism, and charge and spin transport, are very interesting for nanoscale physics. In particular, nanostructures with zigzag edges are expected to have spin-polarized electronic edge states. The synthesized structures could play a leading role in graphene-based spintronics.

**Figure 8:**
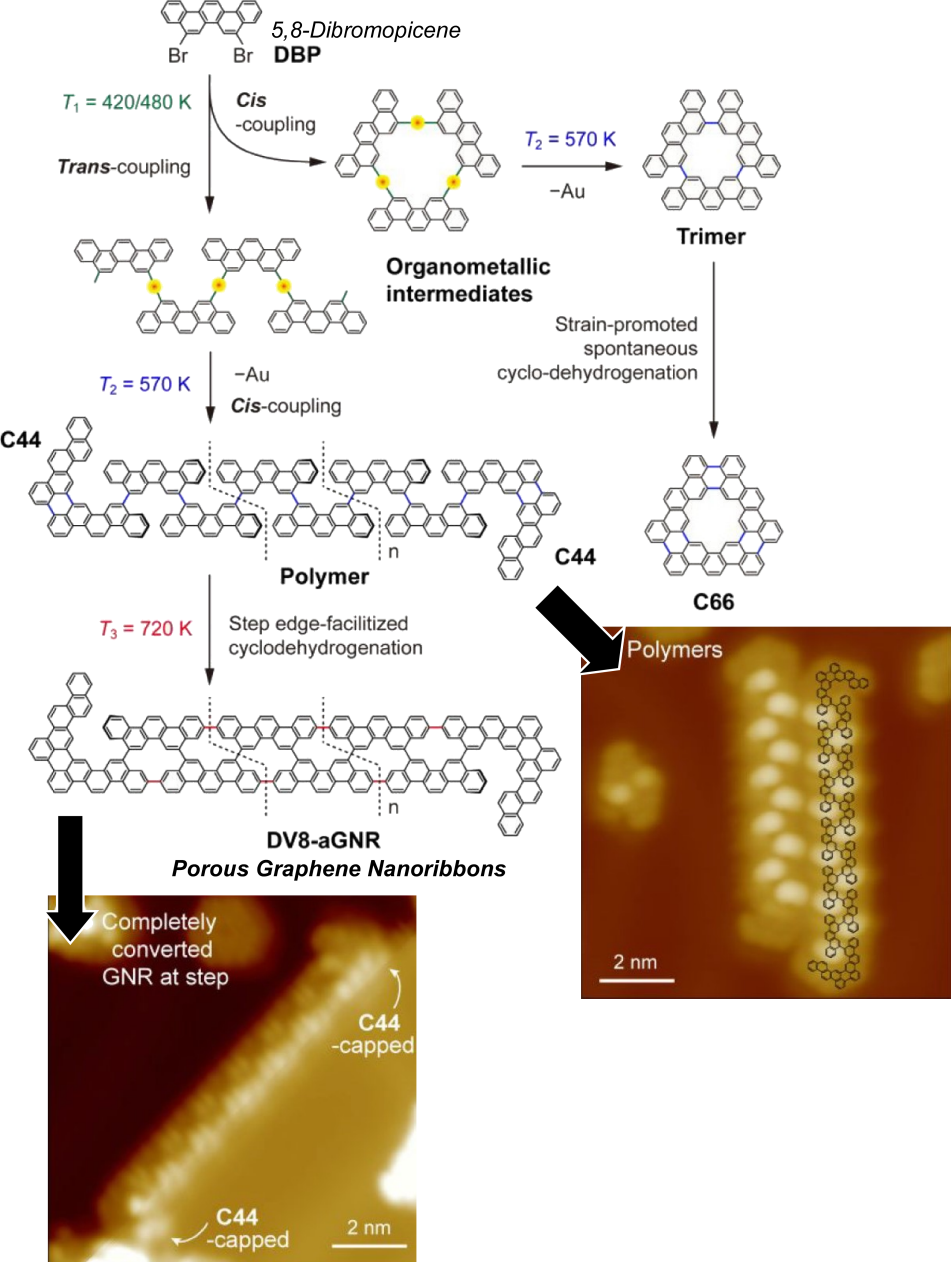
On-surface synthesis of porous graphene nanoribbons on a Au(111) surface. Figure 8 was adapted with permission from [124], Copyright 2022 American Chemical Society. This content is not subject to CC-BY-4.0.

In addition to bandgap engineering of porous graphene nanoribbons via their width and edge arrangement, periodic nanostructures provide a means to control the electronic properties of graphene nanoribbons. Ma, Tan, Wang, and co-workers have synthesized 5,8-dibromopicene on Au(111) surfaces via trans- and cis-coupling to synthesize 8-carbon armchair graphene nanoribbons and nanographene C66 with periodic vacancies on the surface (Figure 8) [124]. Detailed processes of the surface synthesis of 5,8-dibromobenzene molecules kept at room temperature after deposition on Au(111) were disclosed through STM observations. Dehalogenation of some precursor molecules was induced upon annealing at 420/480 K, forming short chains or cyclic organometallic intermediates. In addition to isolated nanographene containing V-shaped C44 and hexagonal C66 on the platform, formation of polymer chains was confirmed after annealing. The conversion of polymer chains to 8-carbon armchair graphene nanoribbons was observed to be efficient at the Au(111) monatomic step at around 670–720 K. The asymmetric distribution of periodic vacancies can form frontier orbitals with wiggly and linear geometries. On-surface synthetic nanoarchitectonics may lead to a variety of nonplanar graphene nanoribbons with periodic atomic vacancies.

**Figure 9:**
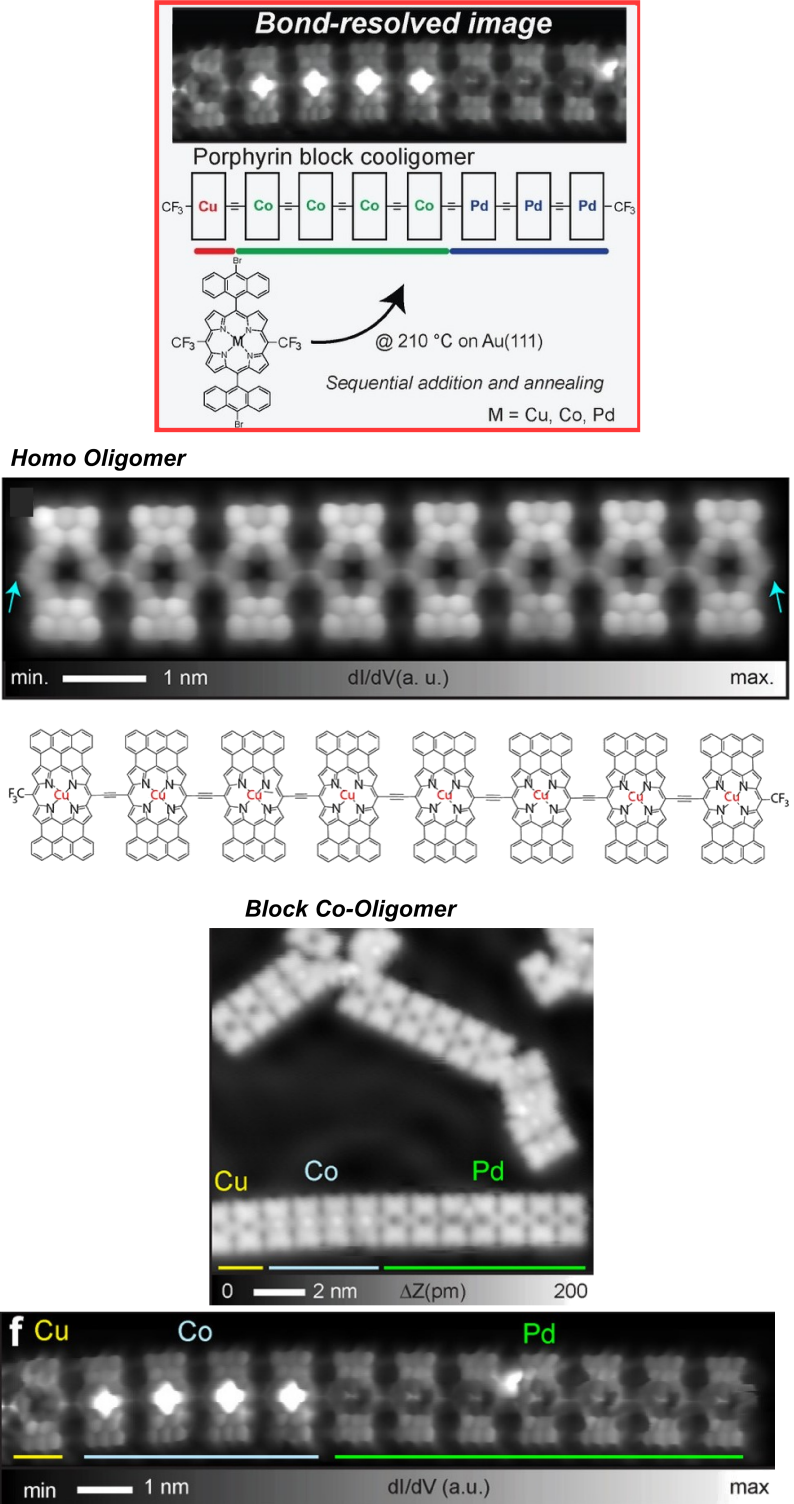
On-surface synthesis of a multi block co-oligomers from trifluoromethyl (CF_3_)-substituted porphyrin metal complexes. Figure 9 was adapted from [125], S. Kawai et al., “On-Surface Synthesis of Porphyrin-Complex Multi-Block Co-Oligomers by Defluorinative Coupling”, Angew. Chem., Int. Ed., with permission from John Wiley and Sons. Copyright © 2021 Wiley-VCH GmbH. This content is not subject to CC-BY-4.0.

Molecular nanoarchitectonics by on-surface synthesis is a powerful tool for structures other than nanographene. Various functional units can be freely combined to obtain new nanostructures. However, it is generally difficult to link units together in a controlled manner, as reactants are often activated simultaneously at a given temperature. Kawai, Ishikawa, Saito, and coworkers have developed a method to synthesize a multiblock copolymer of a porphyrin metal complex on a surface using trifluoromethyl (CF_3_)-substituted 5,15-bis(10-bromoanthracen-9-yl)-10,20-bis(trifluoromethyl)porphyrin precursors (Figure 9) [125]. The ends of the formed oligomer preserve CF_3_ groups after the single-component oligomerization, further enabling sequential block coupling. After annealing the initially built copper porphyrin oligomers at 210 °C, elongated one-dimensional structures were formed, ranging in length from a few nanometers to several tens of nanometers. They still have CF_3_ groups at both ends. The CF_3_ groups undergo coupling reactions at high temperature only in case of sufficiently close approaches of the reactants. Sequential block oligomerization was actually demonstrated using cobalt porphyrin molecules and copper porphyrin oligomers upon annealing again at 210 °C. The block elongation was confirmed through the contrast difference of porphyrin centers (concave and convex), corresponding to Cu and Co porphyrin units, respectively. Next, the authors went one step further and deposited Pd porphyrin molecules on Au(111), where diblock co-oligomers were already present, and annealed them again. It was confirmed that triblock co-oligomers were formed as expected, albeit in relatively small yields. Thus, multiblock oligomers were successfully synthesized by sequential coupling of CF_3_-substituted copper, cobalt, and palladium porphyrins on the surface. The assembly of different porphyrin oligomers combines multiple functional properties such as gas adsorption and magnetism in a one-dimensional structure. This approach could also be a promising strategy to construct designer nanoscale carbon materials by sequentially linking multiple monomers on a surface. It is expected to contribute to future carbon-based nanoelectronics.

**Figure 10:**
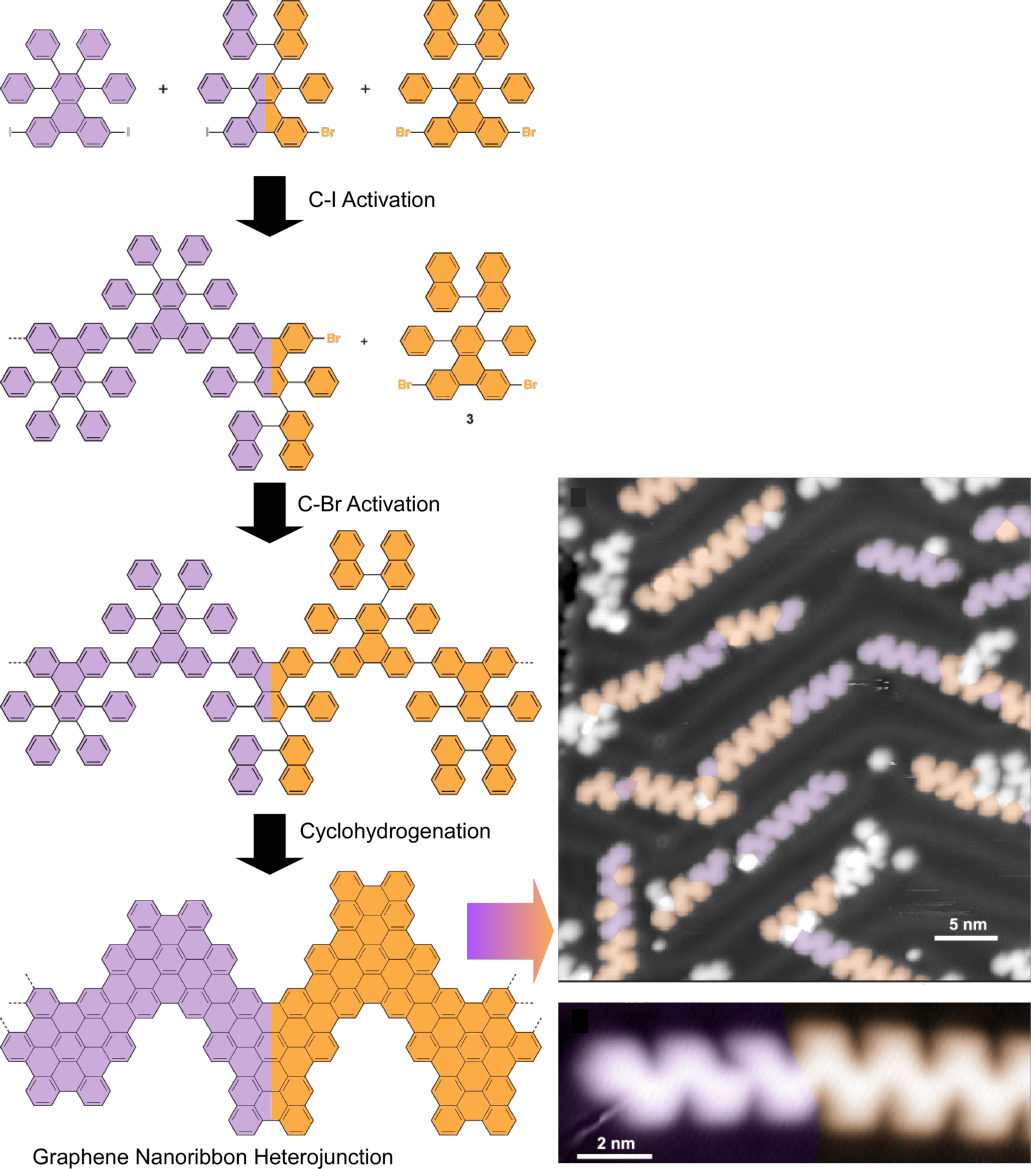
On-surface synthesis of graphene nanoribbons with heterojunction interface upon strategy based on the difference in dissociation energies of C–Br and C–I bonds. Figure 10 was adapted with permission from [126], Copyright 2018 American Chemical Society. This content is not subject to CC-BY-4.0.

Junctions between molecular block units have also been reported with graphene nanoribbons. Nanoarchitectonics of graphene nanoribbon heterojunctions has the potential to be a major technological breakthrough because of the rational design and by virtue of the extraordinary structural and electronic properties of such heterojunctions. However, graphene nanoribbon heterojunction structures made by bottom-up synthesis are usually difficult to incorporate into functional nanodevices because of the random arrangement of the heterojunctions. Bronner, Fischer, Crommie, and co-workers have developed a single hierarchical fabrication strategy via on-surface synthesis of graphene nanoribbons with a single heterojunction interface (Figure 10) [126]. This synthesis strategy is based on the difference in dissociation energies of C–Br and C–I bonds. The growth order of the block copolymers of graphene nanoribbons can be controlled. Such heterojunctions provide a viable platform that can be used directly for functional graphene-nanoribbon-based devices at the molecular scale. The authors used graphene nanoribbon precursors with both ends modified with iodine, graphene nanoribbon precursors with binaphthyl groups modified with bromine at both ends, and graphene nanoribbon linker molecules with iodine and bromine at each end. At lower polymerization temperatures, only the C–I bond is activated, leading to the growth of homopolymers of graphene nanoribbon precursors modified with iodine at both ends, which are terminated by the linker molecules. At higher reaction temperatures, the C–Br bond is cleaved and polymerization of graphene nanoribbon precursors with bromine-modified binaphthyl groups at both ends begins. Cyclohydrogenation at even higher temperatures yields fully cyclized nanographene heterojunctions. The use of bifunctional linker molecules (molecules containing C–I and C–Br bonds) significantly improves single heterojunction yields. The improved structural control of heterojunctions is expected to enable the incorporation of graphene nanoribbon heterostructures with atomic precision in future nanoelectronic devices.

**Figure 11:**
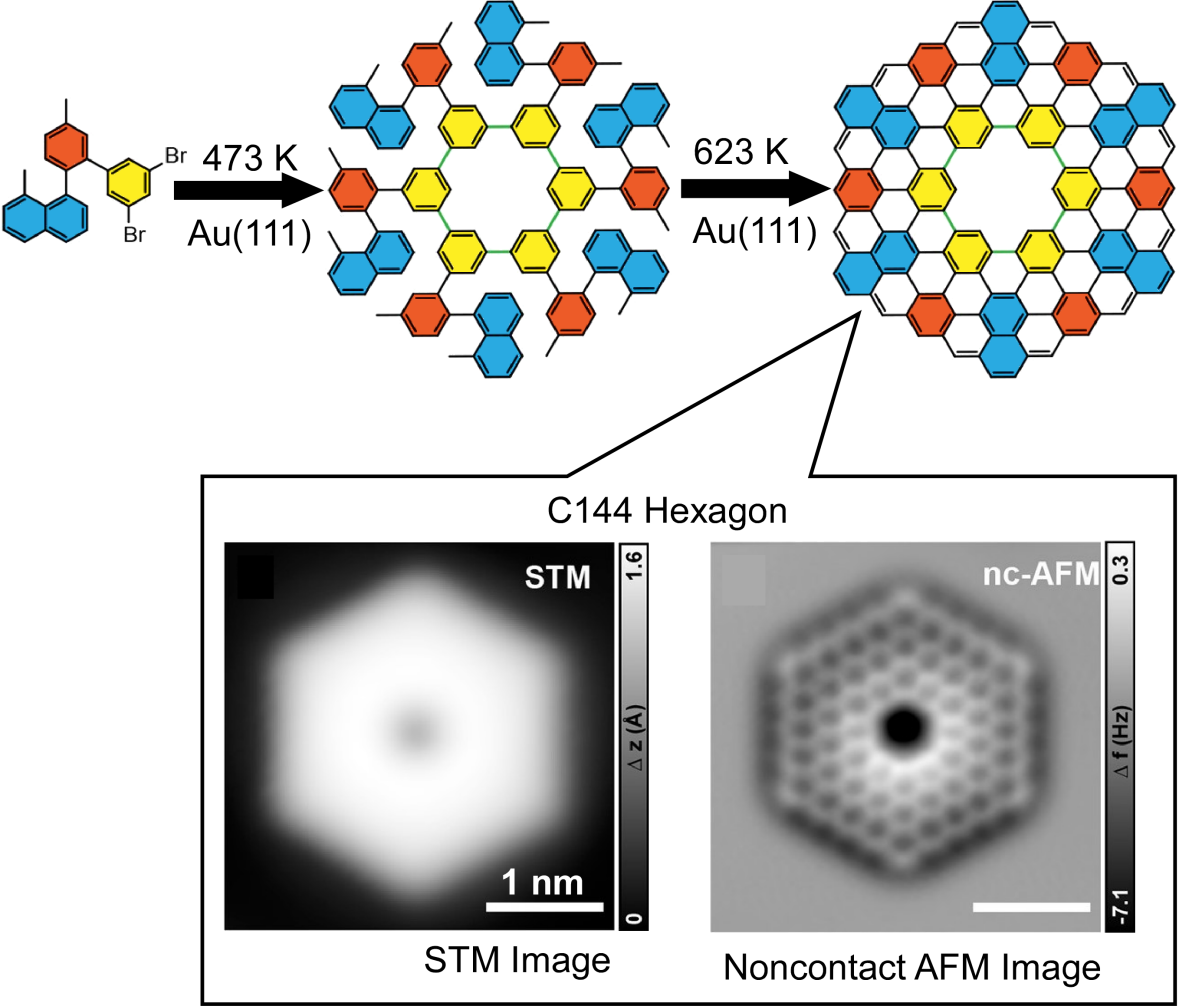
On-surface synthesis of a C144 hexagon with a zigzag outer edge by hierarchical Ullmann coupling and cyclodehydrogenation on a Au(111) surface. Figure 11 was adapted with permission from [127], Copyright 2022 American Chemical Society. This content is not subject to CC-BY-4.0.

Nanocarbons with a defined two-dimensional extent are also attractive targets for on-surface synthetic nanoarchitectonics. Coronoids are compounds possessing both polycyclic aromatic macrocycles and defined cavities. Because of their unique electronic structures, they receive much attention. The electronic properties of these nanoporous graphene-like compounds are modified through changing size and topology of inner and outer rims in their structures. Especially, extended hexagonal coronoids with zigzag outer edges still pose synthetic challenges. Sun, Yan, Yu, and co-workers have successfully synthesized a C144 hexagon with a zigzag outer edge by hierarchical Ullmann coupling and cyclodehydrogenation on a Au(111) surface (Figure 11) [127]. The precursors of this synthesis are methylnaphthalene, methylbenzene, and 3,5-dibromobenzene at 300 K on the Au(111) surface with a thickness less than a single layer. The sample was first annealed at 473 K for 10 min. The bonding of six molecules of the precursor caused macrocyclization, resulting in a polyphenylene dendrimer. Further annealing at 623 K for 2 min resulted in surface-assisted cyclic hydrogenation and eventual conversion to the target zigzag coronoid C144. A magnified STM image of zigzag coronoid C144 reveals a hexagonal graphene nanoflake structure with sixfold symmetry and a central cavity. Fine-tuning of electronic properties by zigzag topology and the development of coronoids with multiple radical properties in open shells are expected.

**Figure 12:**
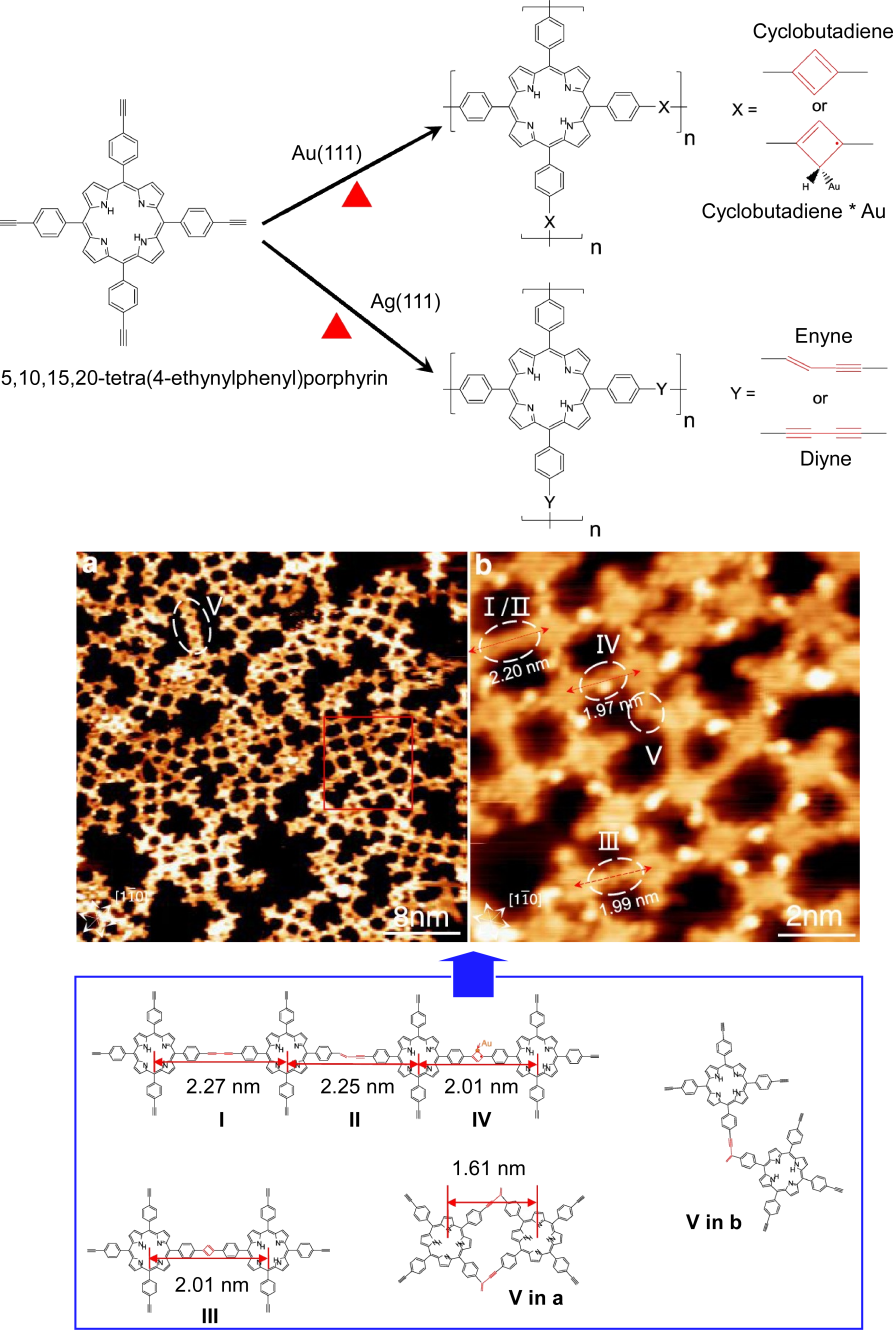
On-surface synthesis of covalent organic frameworks (COFs) from 5,10,15,20-tetra(4-ethynylphenyl)porphyrin molecules via a solid–vapor interfacial reaction. Figure 12 was adapted with permission from [129], Copyright 2021 American Chemical Society. This content is not subject to CC-BY-4.0.

Surface covalent organic frameworks are atomically thick sheets of covalently bonded organic building blocks. Expecting unique properties such as low dimensionality, well-defined in-plane structure, and tunable functionality, Wang, Wan, and co-workers reported a method for the nanoarchitectonics of highly ordered surface covalent organic frameworks (COFs) via a solid–vapor interfacial reaction [128]. In this method, one precursor is vaporized and the other precursor is introduced to the surface in advance, allowing coupling reactions to occur at the solid–vapor interface. Various two-component chemical reactions can be applied to this methodology, not limited to the Schiff base reaction they reported. Thus, rational nanoarchitectonics of single-layer surface COFs with desired functions is expected. The on-surface synthesis of COFs using large molecules such as tetraphenylporphyrin molecules has also been investigated. Wang, Zhong, Ji, and co-workers achieved the on-surface synthesis of COFs from 5,10,15,20-tetra(4-ethynylphenyl)porphyrin molecules (Figure 12) [129]. The reaction mode changes depending on the substrate. Alkyne–alkyne cyclic dimerization is the dominant reaction pathway on Au(111), while Glaser coupling and nonhydrogenic head-to-head coupling are the dominant reaction pathways on Ag(111). In the reaction n Ag(111), the molecules are alternately arranged and tightly packed. Rectangular and elliptical pores exist between adjacent molecular rows. Reactions occurred first in the rectangular pores. This is because the possible reaction pathways are greatly reduced in the two-dimensional solid phase, and the terminal alkynes of neighboring molecules with rectangular pores are closer together, facilitating the reaction. These techniques allow for the nanoarchitectonics of two-dimensional COFs.

Although the abovementioned examples describe only a part of the past accomplishments, connecting molecules, building up molecules, and organizing molecules can now be accompanied by directly observing the structures. This is due to significant development in nanotechnology that can alter basic thinking about traditional synthetic organic chemistry. Nanotechnological tools can also be used to induce site-specific organic synthesis and alter selectivity, as seen in the example of local probe chemistry. Indeed, it is now possible to pick up specific substrate molecules and make them react at desired molecular sites. This could be the starting point for assembling functional materials from the molecular level. Hence, molecular nanoarchitectonics is a fusion of advanced nanotechnology and conventional organic chemistry.

## Perspectives

In this review, we have presented several examples of what we consider to be molecular nanoarchitectonics, with typical examples of research in local probe chemistry and on-surface synthesis. As shown in these examples, nanotechnology has made great contributions to organic chemistry, and it is now possible to observe reaction processes at molecular resolution. In addition, organic synthesis can be freely controlled by manipulation at the molecular level using probe tips. These results show that it is possible to freely synthesize substances at the molecular level. These studies also show that local physical properties can be analyzed by measurements with very high resolution. Thus, it is possible to observe organic chemical reactions at the atomic and molecular level and to understand the properties of molecules at that level.

The current mainstream of functional material fabrication is a combination of basic technologies such as organic synthesis, self-assembly, or structure fabrication methods such as the Langmuir–Blodgett (LB) method [130–131] or layer-by-layer (LbL) assembly [132–134]. These conventional methodologies are now being used to create materials that can address social issues such as energy requirements [135–136], the environment [137–138], and medicine [139–140]. Many analytical studies have shown that high effectiveness and activity are due to features at the atomic and molecular level [141–142]. It is often observed that a particular molecular structure or an atom at a particular site can be highly functional and active. Molecular nanoarchitectonics, as shown here, can be an important key to understand such aspects and to create high-performance materials in a rational way. The linkage of nanotechnology and fundamental science such as organic synthesis can be the cornerstone of materials nanoarchitectonics as a whole.

This approach to molecular nanoarchitectonics based on high-resolution observation is a field rooted in basic science. The fusion of this basic science field with the field of pursuing the application of practical functional materials will complete materials chemistry for everything. The choice of materials is diverse and unlimited. In order to position nanoarchitectonics as a method for everything and as a completed form of general materials science, it is necessary to consider a wide range of applications and many combinations and possibilities. In the past, it has been difficult to apply the method to a seemingly infinite number of substances. However, the development of computational methods such as artificial intelligence will make this possible in current science [143–144]. In fact, the fusion of nanoarchitectonics and materials informatics has been proposed [145]. Nanotechnology worked as a game changer. Nanoarchitectonics integrates nanotechnology and traditional sciences to a more unified materials science. Completion of materials science by nanoarchitectonics will be accelerated with the aid of computer science and technology.
